# Real-world effectiveness and safety of 200 mg abrocitinib initiation in Chinese adults with moderate-to-severe atopic dermatitis: a retrospective study

**DOI:** 10.3389/fimmu.2026.1789298

**Published:** 2026-05-15

**Authors:** Peilin Lu, Judan Zhong, Na Tan, Aijun Chen, Tao Cai, Jin Chen, Shuang Chen

**Affiliations:** 1 Department of Dermatology, The First Affiliated Hospital of Chongqing Medical University, Chongqing, China; 2 Department of Dermatology, The Affiliated Hospital, Southwest Medical University, Luzhou, Sichuan, China

**Keywords:** abrocitinib, adverse events, atopic dermatitis, effectiveness, JAK inhibitor, real-world evidence

## Abstract

**Background:**

Abrocitinib, a Janus kinase (JAK) inhibitor approved for moderate-to-severe atopic dermatitis (AD), has demonstrated efficacy at 100 mg in randomized controlled trials. However, real-world data on 200 mg initiation in Chinese patients remain scarce.

**Objective:**

To evaluate the real-world effectiveness and safety of initiating abrocitinib at 200 mg in Chinese adults with moderate-to-severe AD.

**Methods:**

This retrospective study included 20 patients with moderate-to-severe AD treated with abrocitinib. Patients were stratified into Group A (n=7, initiated at 200 mg, tapered to 100 mg if EASI-75 or ≥4-point PP-NRS reduction was achieved by week 12) and Group B (n=13, initiated at 100 mg, escalated to 200 mg if targets were not met). Primary outcomes included Eczema Area and Severity Index (EASI), Investigator Global Assessment (IGA), Peak Pruritus Numeric Rating Scale (PP-NRS), and Dermatology Life Quality Index (DLQI). Adverse events (AEs) were recorded.

**Results:**

Both groups showed significant reductions in all outcome measures at weeks 4, 12, and 24. Baseline DLQI was significantly higher in Group A (22.0 vs. 18.0, P < 0.001). At week 4, Group A showed superior PP-NRS improvement (P = 0.019). By week 12, Group A achieved significantly higher rates of EASI < 5 (57.14% vs. 0%, P < 0.01) and IGA 0/1 (85.71% vs. 7.69%, P < 0.01). Notably, 50% of patients had failed prior systemic therapies, including 85.7% in Group A, reflecting the refractory nature of the cohort. At week 24, Group A maintained superiority in EASI and PP-NRS (P < 0.05), while EASI-75 response exceeded 90% in both groups. AEs were comparable between groups (71.43% vs. 61.54%), with no serious events.

**Conclusion:**

The 200 mg initiation strategy offers rapid and sustained disease control, particularly for patients with high baseline disease activity and severe quality of life impairment. The 100 mg strategy achieves favorable outcomes by week 24 for those with milder burden. These findings support personalized dosing, though larger prospective studies are warranted to confirm results.

## 1 Introduction

Atopic dermatitis (AD) is a common inflammatory skin disease characterized by chronic pruritus and recurrent eczema, substantially impacting quality of life. Epidemiology indicates a global prevalence of 1.7%–32.8% in children and 1.2%–9.7% in adults (1, 2). While systemic therapies such as cyclosporine A, corticosteroids, and methotrexate are used for moderate-to-severe AD, their long-term utility is often limited by modest efficacy or safety concerns (3–5).

The pathogenesis of AD involves complex interactions between skin barrier dysfunction and immune dysregulation, with cytokine signaling mediated by the Janus kinase (JAK)/Signal Transducer and Activator of Transcription (STAT) pathway playing a pivotal role (6). Abrocitinib, a highly selective JAK1 inhibitor approved in 2022, has demonstrated significant efficacy in multiple clinical trials (7–11).

Current guidelines recommend initiating abrocitinib at 100 mg, escalating to 200 mg if efficacy is inadequate. However, clinicians often face a dilemma: whether to start with a higher dose for rapid relief or a standard dose to minimize potential adverse effects. Early identification of patients likely to have a suboptimal response to standard dosing, and the evaluation of a 200 mg induction strategy in a real-world setting, are critical for clinical decision-making. This retrospective study analyzes data from patients with moderate-to-severe AD treated with abrocitinib in our department to compare the Effectiveness and safety of 100 mg versus 200 mg initiation strategies, aiming to provide a reference for individualized management.

## 2 Materials and methods

### 2.1 Study design

This was a single-center, retrospective, non-randomized, real-world observational study. We enrolled patients with moderate-to-severe AD who visited our hospital between January 2023 and December 2024 and received abrocitinib (with exposure to 200 mg within the 24-week treatment period). The study protocol was approved by the Ethics Committee of The First Affiliated Hospital of Chongqing Medical University (No. KX2025-KYC0034-01).

### 2.2 Inclusion criteria

Included patients met all of the following criteria:

1. Age ≥18 years;
2. Diagnosis of AD confirmed by clinical diagnosis based on the Williams diagnostic criteria (12);
3. Moderate-to-severe severity, defined as Investigator’s Global Assessment (IGA) score ≥ 3, Eczema Area and Severity Index (EASI) score ≥ 16, or affected body surface area (BSA) ≥ 10%, and Pruritus Numerical Rating Scale (PP-NRS) score ≥ 4;
4. Inadequate response or intolerance to topical therapies (corticosteroids or calcineurin inhibitors).

### 2.3 Exclusion criteria

Patients were excluded if they met any of the following conditions:

1. Total duration of abrocitinib treatment was less than 24 weeks;
2. Had active hepatitis B, tuberculosis, malignant tumors, thrombotic diseases, or severe psychiatric disorders.
3. Patients with active hepatitis B, tuberculosis, malignancy, thrombotic diseases, or severe psychiatric history were excluded.

### 2.4 Dosing strategy and group allocation

According to the standardized diagnosis and treatment protocol of our department, patients were allocated into two groups based on the initial dose and subsequent dose adjustment strategy: Group A (Step-down Strategy): Initial dose of 200 mg/day. If patients achieved EASI-75 response (EASI score reduction ≥ 75% from baseline) or a > 4-point reduction in PP-NRS score at Week 12, the dose was tapered to 100 mg/day and maintained until Week 24; Group B (Step-up Strategy): Initial dose of 100 mg/day. If patients did not achieve the above-mentioned treatment targets at Week 12, the dose was escalated to 200 mg/day and continued until Week 24. During the 24-week treatment period, patients did not receive any concomitant systemic or topical therapies for atopic dermatitis. This included topical corticosteroids, topical calcineurin inhibitors, oral antihistamines, and other immunosuppressive agents.

### 2.5 Data collection and assessment

Baseline demographic and clinical data were collected, including age, sex, body mass index (BMI), personal and family history of atopic diseases (e.g., asthma, allergic rhinitis) and allergies. Disease severity was evaluated using EASI, IGA, PP-NRS, and Dermatology Life Quality Index (DLQI) at baseline, Week 4, Week 12, and Week 24. Laboratory and imaging examinations were performed at baseline to screen for contraindications, including peripheral eosinophil count, serum total immunoglobulin E (IgE) level, liver and renal function, coagulation profile, T-cell spot test for tuberculosis (T-SPOT.TB), and chest X-ray. Adverse events (AEs) were monitored throughout the 24-week treatment period. The occurrence, clinical manifestations, severity, management measures, and outcomes of all AEs were recorded in detail. All data were collected from a dedicated atopic dermatitis specialty clinic at our institution. Specialized physicians and coordinators managed patient follow-up. Follow-up visits were scheduled at Weeks 4, 12, and 24, with a permissible window of ±3 days. Patients who missed the scheduled visit window were excluded from the analysis. This standardized protocol ensured complete data collection at the specified time points for all included patients.

### 2.6 Statistical analysis

Statistical analyses and graphing were performed using IBM SPSS Statistics 27.0 and GraphPad Prism 10.0. A two-tailed P-value < 0.05 was considered statistically significant. The normality of continuous variables was assessed using the Shapiro-Wilk test. Data were expressed as mean ± standard deviation (SD) for normally distributed variables, or median with interquartile range (IQR, Q1–Q3) for non-normally distributed variables. Between-group comparisons were conducted using the independent Student’s t-test or the Mann-Whitney U test (exact test), as appropriate. For within-group comparisons across multiple time points, the Friedman test was employed, followed by *post hoc* pairwise comparisons using the Wilcoxon signed-rank test with Bonferroni correction. Categorical data were presented as percentages (95% confidence interval [CI]). Within-group comparisons were analyzed using Cochran’s Q test and the corrected McNemar test. Longitudinal differences between groups were assessed using Generalized Estimating Equations (GEE) models, supplemented by Chi-square tests for comparisons at individual time points. To assess the clinical relevance of improvements of DLQI, the minimal clinically important difference (MCID) was applied and set at 4 points, indicating that a reduction of ≥4 points in DLQI score represents a clinically meaningful improvement in patients’ quality of life (13).

## 3 Results

### 3.1 Study population

A total of 20 patients with moderate-to-severe atopic dermatitis who received 200 mg abrocitinib were included in this study. The baseline demographic and clinical characteristics are summarized in **Table 1**. Females accounted for 40.0% (8/20) of the cohort. The mean age was 34.50 ± 9.65 years, and the median disease duration was 4.5 (IQR: 2.0–13.5) years. Regarding comorbidities, 35% (7/20) of patients had allergic rhinitis, 10% (2/20) each had asthma, conjunctivitis, or food allergy, and 5% (1/20) had contact dermatitis. All patients had previously received conventional topical treatments and antihistamines.

**Table 1:** Baseline demographic and clinical characteristics.

Characteristic	Total (A+B, n=20)	Group A (n=7)	Group B (n=13)	*p*
General data				
Age, years (median, IQR)	31.5 (26.5, 40.0)	40.0 (32.5, 40.0)	29.0 (27.0, 35.0)	0.588
BMI, kg/m2 (median, IQR)	24.4 (22.4, 26.8)	22.9 (22.6, 24.7)	25.2 (22.4, 27.3)	0.438
Female gender, n (%)	8 (40.0)	4 (57.1)	4 (30.8)	0.356
Disease duration	4.5 (2.0, 13.5)	6.0 (3.50 12.5)	3.0 (2.0,11.0)	0.536
Baseline severity				
EASI (median, IQR)	20.0 (17.9,25.1)	23.1 (19.0,36.8)	18.6 (17.2,24.1)	0.241
IGA (median, IQR)	4.0 (4.0, 4.0)	4.0 (3.0,4.0)	4.0 (4.0, 4.0)	0.643
IGA=4, n (%)	16 (80.0)	5 (71.4)	11 (84.6)	0.587
PP-NRS (median, IQR)	7.5 (6.0, 9.0)	8.0 (6.0, 9.0)	7.0 (6.0, 8.5)	0.485
DLQI (median, IQR)	20.0 (17.0, 21.0)	22.0 (21.0,24.0)	18.0 (17.0,20.0)	**<0.001**
Previous systematic therapy, n (%)	10 (50.0)	6 (85.7)	4 (30.8)	0.057
dupilumab	4 (20.0)	3 (42.9)	1 (7.7)	0.101
baricitinib	1 (5.0)	1 (14.3)	0 (0.0)	0.350
tofacitinib	5 (25.0)	3 (42.9)	2 (15.4)	0.290
upadacitinib	2 (10.0)	1 (14.3)	1 (7.7)	1.000
*radix tripterygil*	1 (5.0)	0 (0.0)	1 (7.7)	1.000
Associated conditions, n (%)	10 (50.0)	4 (57.1)	6 (46.2)	1.000
rhinitis	7 (35.0)	3 (42.9)	4 (30.8)	0.651
conjunctivitis	2 (10.0)	2 (28.6)	0 (0.0)	0.111
asthma	2 (10.0)	1 (14.3)	1 (7.7)	1.000
food allergy	2 (10.0)	2 (28.6)	0 (0.0)	0.111
allergic dermatitis	1 (5.0)	0 (0.0)	1 (7.7)	1.000
atopic family history	7 (35.0)	2 (28.6)	5 (38.5)	1.000

BMI, Body Mass Index; DLQI, Dermatology Life Quality Index; EASI, Eczema Area and Severity Index; PP-NRS, Peak Pruritus Numerical Rating Scale; IGA, Investigator Global Assessment; SD, Standard Deviation; IQR, Interquartile Range; n, number. P < 0.05 was considered significant. Only DLQI was statistically significant (highlighted in bold).

Prior to abrocitinib initiation, 50% (10/20) of patients had been treated with other systemic agents, including tofacitinib (n=5), dupilumab (n=4), upadacitinib (n=2), baricitinib (n=1), and *radix tripterygil* (n=1). Abrocitinib therapy was initiated due to severe skin lesions (IGA >3), intense pruritus (PP-NRS >6), and/or inadequate response to prior systemic therapies.

Patients were stratified into two groups based on the initial dosage: Group A (200 mg/day, n=7, 35%) and Group B (100 mg/day, n=13, 65%). At baseline, overall disease activity was high, with a median EASI score of 20.0(17.9,25.1), a median IGA score of 4.0 (IQR: 4.0–4.0), and a median PP-NRS score of 7.5 (IQR: 6.0–9.0). Between-group comparisons revealed no statistically significant differences in most general data, baseline severity, previous systematic therapy, and associated condtions(all p > 0.05), indicating baseline comparability. However, baseline DLQI scores differed significantly between groups: Group A had a higher score (22.0 vs. 18.0, p < 0.001), with the difference exceeding the MCID threshold (4 points). This suggests that patients in Group A experienced more severe quality of life impairment at baseline. Additionally, the number of prior systemic therapies showed a marginal between-group difference (p = 0.057). Neither group received additional treatments, so there were no between-group differences in concomitant medication use.

### 3.2 Effectiveness

**Figure 1:**
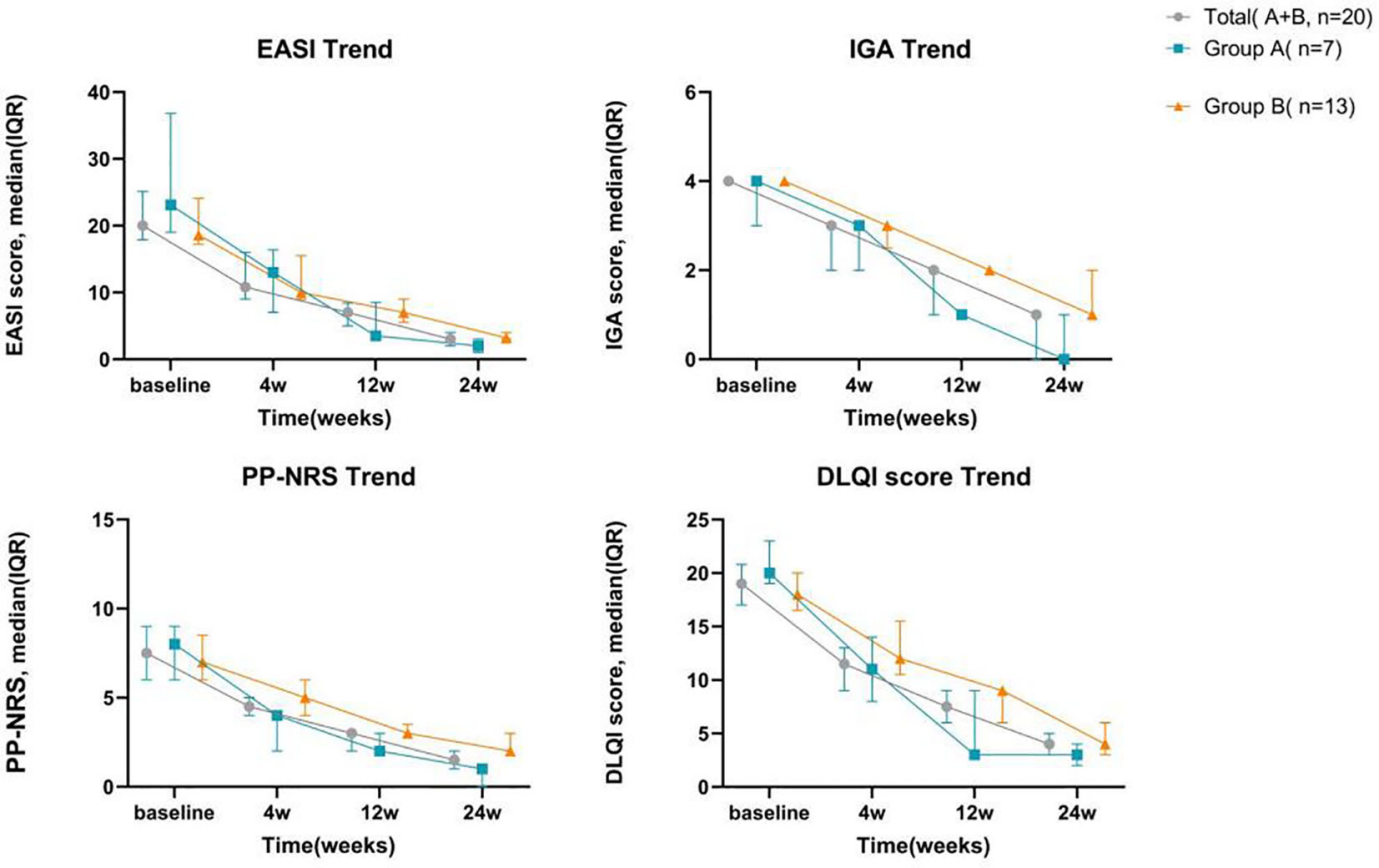
Changes of follow-up indicators with the course of treatment IQR, the interquartile range.

Overall, the scores for EASI, IGA, PP-NRS, and DLQI exhibited a consistent downward trend throughout the treatment period (**Figure 1**).

#### 3.2.1 EASI scores and response rates (EASI-75, EASI-90)

**Figure 2:**
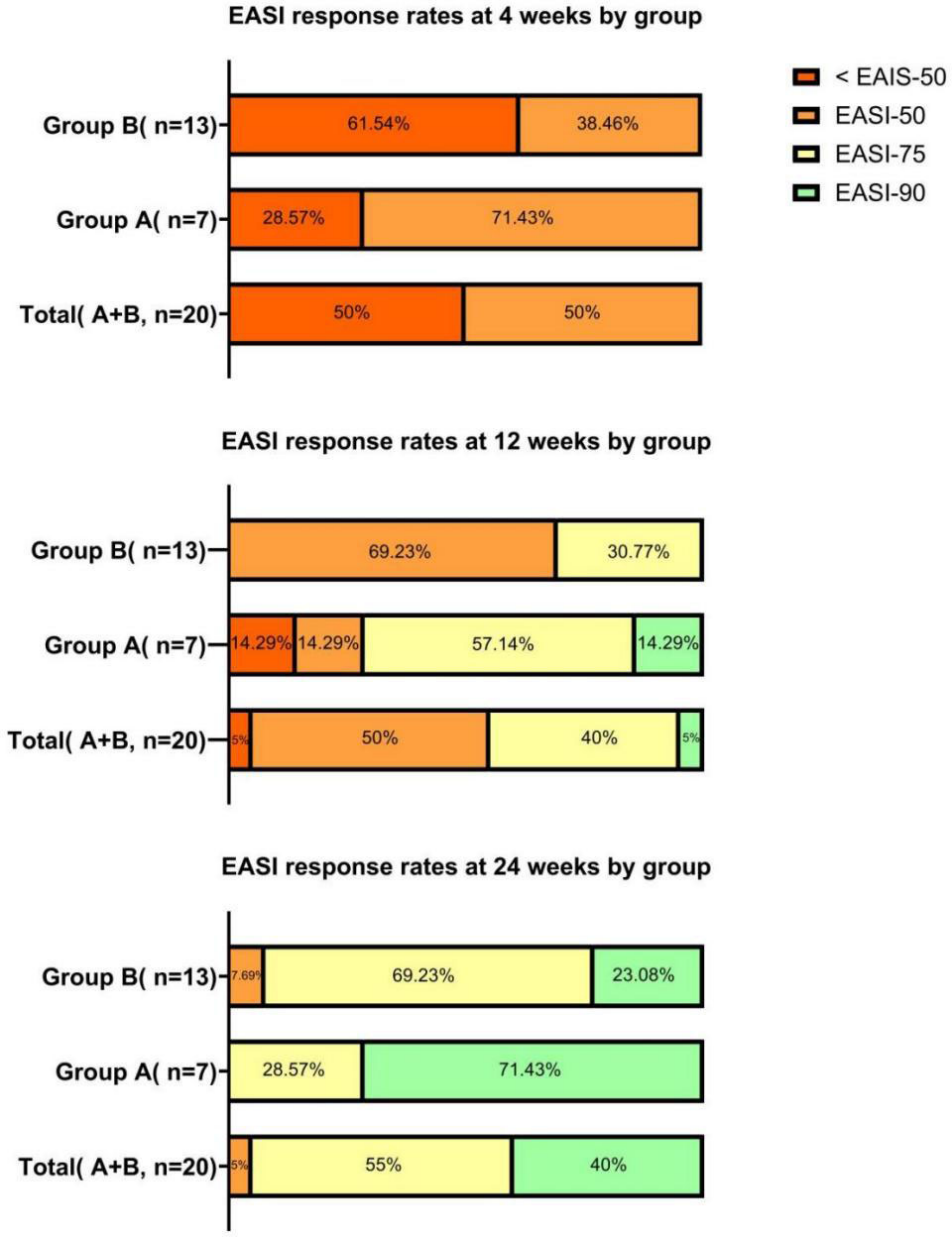
Proportion of patients achieving EASI-50, EASI-75, and EASI-90 responses at weeks 4, 12, and 24.

**Figure 3:**
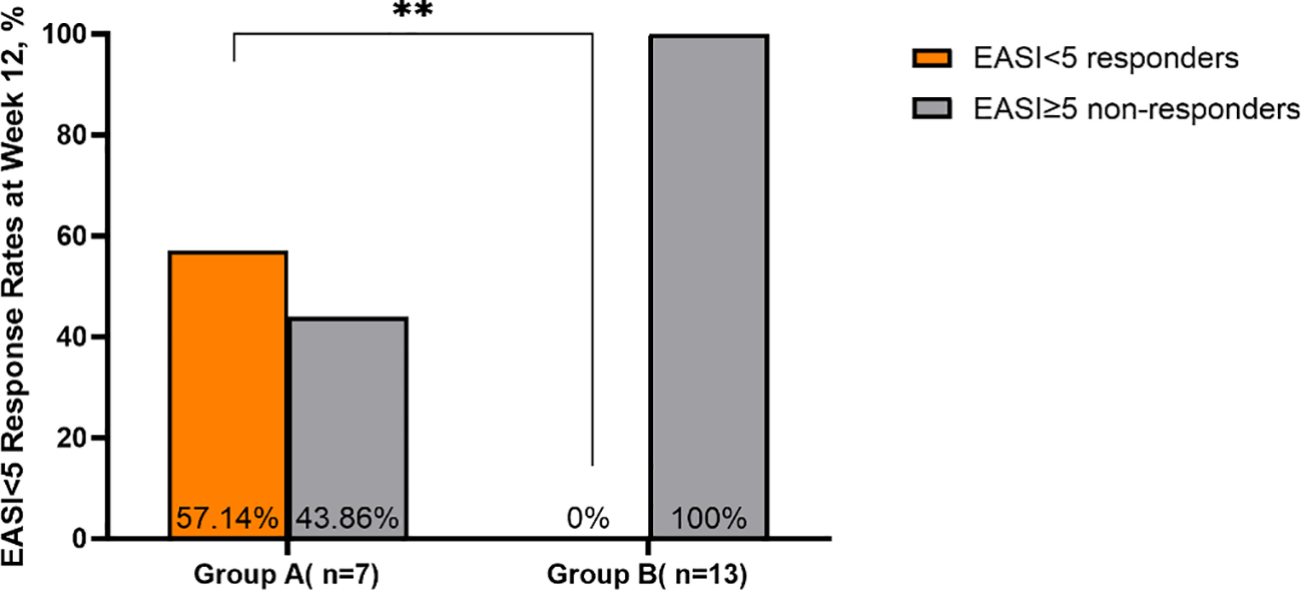
Comparison of EASI < 5 response rates between Group A and Group B at week 12. **p <0.01.

Intragroup comparisons revealed significant differences in EASI scores across the four time points for the total cohort, Group A, and Group B (Total: χ² = 57.840, P < 0.001; Group A: χ² = 19.971, P < 0.001; Group B: χ² = 37.892, P < 0.001), indicating a continuous decline in scores over time. The median EASI score for the total cohort decreased significantly from 20.0 (17.9, 25.1) at baseline to 3.0 (2.0, 4.0) at week 24 (P < 0.001), representing an 85% reduction. Both Group A and Group B showed statistically significant reductions from baseline at all follow-up time points (all P < 0.05). Regarding intergroup comparisons, baseline EASI scores were comparable between groups (P = 0.241). At week 4, the difference between groups was not statistically significant (P = 0.669). However, by week 12 and week 24, EASI scores in Group A were significantly lower than those in Group B (Week 12: P = 0.030; Week 24: P = 0.046), suggesting that Group A maintained a significant and sustained advantage in lesion clearance (**Table 2****;** **Figures 2**, **3**).

**Table 2:** EASI, IGA, PP-NRS, DLQI and statistics analysis of patients in different treatment groups at Week 4, 12, and 24.

Indicator	Group/analysis dimension	Baseline (median, IQR)	Week 4 (median, IQR)	Week 12 (median, IQR)	Week 24 (median, IQR)	Within-group time trend analysis (Friedman test, χ²/df/P-value)
EASI	Overall (A+B, n=20)	20.0 (17.9,25.1)	10.8 (9.0,16.0)	8.0 (5.0,9.0)	3.0 (2.0,4.0)	57.840/3/<0.001
	Within-group difference (paired Wilcoxon test)	–	Z=-3.921, P<0.001	Z=-3.920, P<0.001	Z=-3.920, P<0.001	
	Group A (n=7)	23.1 (19.0,36.8)	13.0 (7.0,16.4)	3.0 (3.0,6.5)	2.0 (1.0,3.0)	21.000/3/<0.001
	Within-group difference (paired Wilcoxon test)	–	Z=-2.366, P = 0.018	Z=-2.366, P = 0.018	Z=-2.366, P = 0.018	
	Group B (n=13)	18.6 (17.2,24.1)	10.0 (9.0,15.5)	8.5 (7.8,9.0)	3.2 (3.0,4.0)	37.698/3/<0.001
	Within-group difference (paired Wilcoxon test)	–	Z=-3.182, P = 0.001	Z=-3.180, P = 0.001	Z=-3.182, P = 0.001	
	Between-group difference (Mann-Whitney U test)	U=30,Z=1.228, P = 0.241,r=0.275	u=40,Z=0.436, P = 0.699,r=0.097	U=73,Z=2.192, P **=** **0.030**,r=0.49	U=71,Z=2.063, P **=** **0.046**,r=0.461	–
IGA	Overall (A+B, n=20)	4.0 (4.0, 4.0)	3.0 (2.0,3.0)	2.0 (1.0,2.0)	1.0 (0,1.0)	57.185/3/<0.001
	Within-group difference (paired Wilcoxon test)	—	Z=-4.119, P<0.001	Z=-3.904, P<0.001	Z=-3.982, P<0.001	
	Group A (n=7)	4.0 (3.0,4.0)	3.0 (2.0,3.0)	1.0 (1.0,1.0)	0 (0,1.0)	19.219/3/<0.001
	Within-group difference (paired Wilcoxon test)	–	Z=-2.271, P = 0.023	Z=-2.333, P = 0.020	Z=-2.414, P = 0.016	
	Group B (n=13)	4.0 (4.0, 4.0)	3.0 (2.5,3.0)	2.0 (2.0,2.0)	1.0 (0,1.0)	38.143/3/<0.001
	Within-group difference (paired Wilcoxon test)	–	Z=-3.500, P<0.001	Z=-3.307, P<0.001	Z=-3.247, P<0.001	
	Between-group difference (Mann-Whitney U test)	Z=-0.685, P = 0.643	Z=-0.897, P = 0.485	Z=-2.672, P **=** **0.014**	Z=-1.119, P = 0.351	—
PP-NRS	Overall (A+B, n=20)	7.5 (6.0, 9.0)	4.5 (4.0,5.0)	3.0 (2.0,3.0)	1.5 (1.0,2.0)	55.607/3/<0.001
	Within-group difference (paired Wilcoxon test)	–	Z=-3.941, P<0.001	Z=-3.860, P<0.001	Z=-3.940, P<0.001	
	Group A (n=7)	8.0 (6.0, 9.0)	4.0 (2.0,4.0)	2.0 (2.0,3.0)	1.0 (0,1.0)	19.103/3/<0.001
	Within-group difference (paired Wilcoxon) test	–	Z=-2.392, P = 0.017	Z=-2.371, P = 0.018	Z=-2.371, P = 0.018	
	Group B (n=13)	7.0 (6.0, 8.5)	5.0 (4.0,6.0)	3.0 (3.0,4.0)	2.0 (1.0,2.0)	36.070/3/<0.001
	Within-group difference (paired Wilcoxon test)	–	Z=-3.211, P = 0.001	Z=-3.129, P = 0.002	Z=-3.200, P = 0.001	
	Between-group difference (Mann-Whitney U test)	Z=-0.771, P = 0.485	Z=2.392, **P** **=** **0.019**	Z=2.650, **P** **=** **0.011**	Z=2.418, **P** **=** **0.024**	—
DLQI	Overall (A+B, n=20)	20.0 (17.0,21.0)	11.5 (10.0,14.0)	7.5 (6.0,9.0)	4.0 (3.0,5.0)	52.096/3/<0.001
	Within-group difference (paired Wilcoxon test)	–	Z=3.925, P<0.001	Z=3.930, P<0.001	Z=-3.924, P<0.001	
	Group A (n=7)	22.0 (21.0,24.0)	11.0 (9.0,13.5)	6.0 (4.5,8.0)	3.0 (2.0,4.0)	19.324/3/<0.001
	Within-group difference (paired Wilcoxon test)	–	Z=2.375, P = 0.018	Z=2.371, P = 0.018	Z=2.371, P = 0.018	
	Group B (n=13)	18.0 (17.0,20.0)	12.0 (11.0,15.0)	8.0 (7.0,10.0)	4.0 (3.0,5.0)	36.969/3/<0.001
	Within-group difference (paired Wilcoxon test)	–	Z=3.189, P = 0.001	Z=3.205, P = 0.001	Z=3.187, P = 0.001	
	Between-group difference (Mann-Whitney U test)	Z=-3.513, **P<0.001**	Z=1.118, P = 0.275	Z=1.727, P = 0.097	Z=-1.699, P = 0.097	—

EASI, Eczema Area and Severity Index; IGA, Investigator’s Global Assessment; PP-NRS, Peak Pruritus Numerical Rating Scale; DLQI, Dermatology Life Quality Index; IQR, Interquartile Range.

Group definitions: Group A: Step-down strategy (initial 200 mg abrocitinib, tapered to 100 mg at Week 12 if EASI-75 or PP-NRS reduction >4 points was achieved); Group B: Step-up strategy (initial 100 mg abrocitinib, escalated to 200 mg at Week 12 if treatment targets were not met).

Statistical methods: Within-group changes: Paired Wilcoxon signed-rank test (vs. baseline), reported as Z value and P value; Between-group comparisons: Mann-Whitney U test, reported as U value, standardized Z value, effect size r (rank-biserial correlation), and P value; Time trend analysis: Friedman test, reported as χ², df, and P value; P-values <0.05 were considered statistically significant (highlighted in bold and underlined).

#### 3.2.2 IGA scores and IGA 0/1 response rates

Similar to the trend observed in EASI scores, IGA scores demonstrated a significant decline over time in the total cohort and within both groups (all P < 0.001; **Table 2**). Baseline IGA scores were comparable between groups (P = 0.643). At week 12, IGA scores in Group A were significantly lower than those in Group B (P = 0.014), whereas no significant intergroup differences were found at weeks 4 and 24 (**Table 2**).

**Figure 4:**
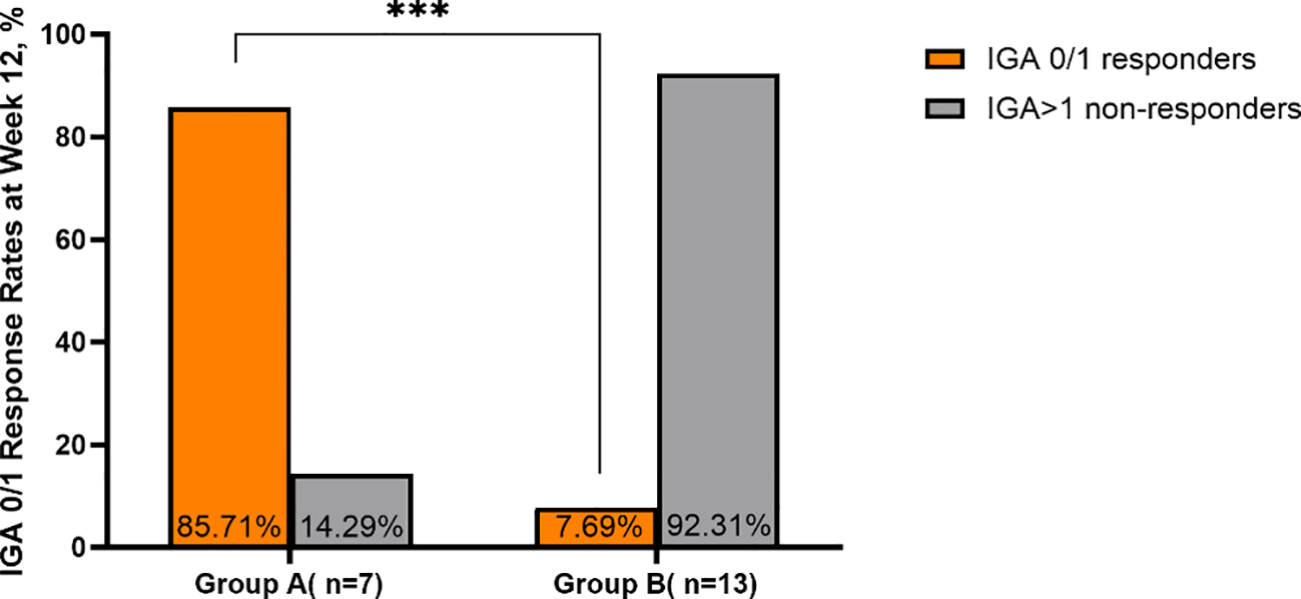
Comparison of IGA 0/1 response rates between Group A and Group B at week 12. ***p < 0.001.

**Figure 5:**
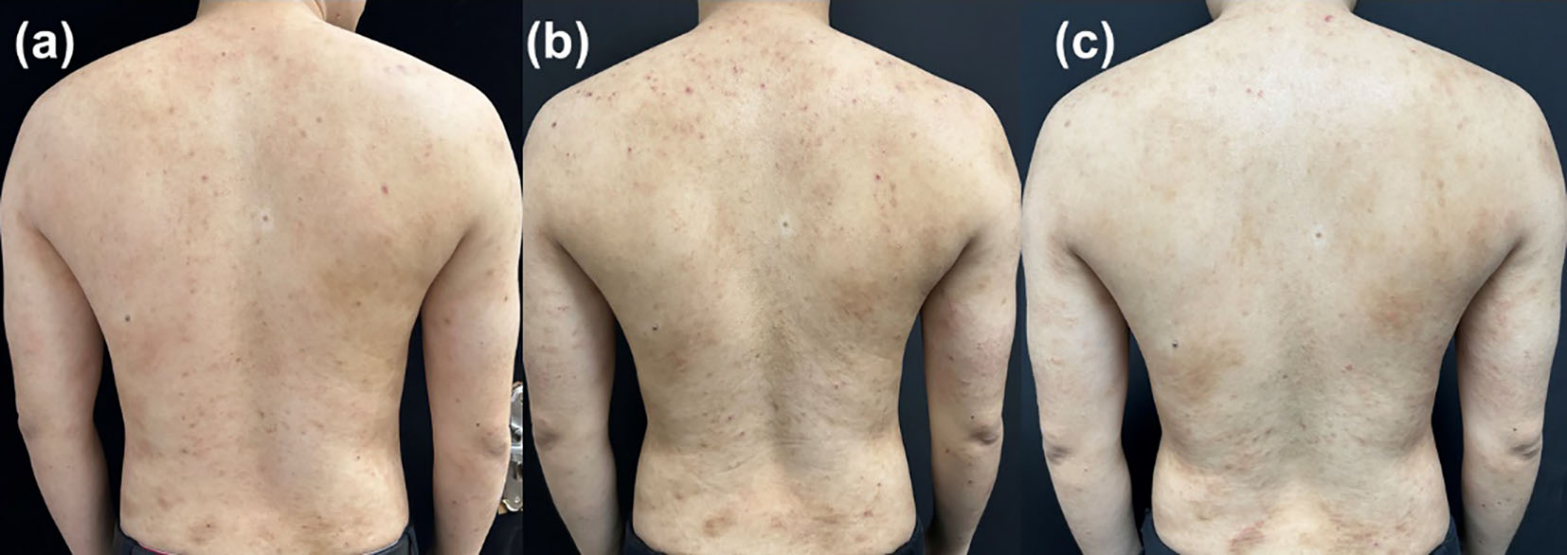
Representative clinical photographs of a patient in group A at **(A)** baseline, **(B)** week 4, **(C)** week 24.

Regarding treatment response, the IGA 0/1 response rate at week 12 was significantly higher in Group A compared to Group B (85.71% vs. 7.69%, P = 0.001). This suggests that initiating treatment with 200 mg abrocitinib can achieve complete or almost complete skin clearance more rapidly (**Table 3****;** **Figures 4**, **5**).

**Table 3:** Number and proportion of patients achieving IGA0/1 in different treatment groups at week 4, 12, and 24.

Treatment group	Sample size (n)	IGA0/1 status at week 4 (n/%)	IGA0/1 status at week 12 (n/%)	IGA0/1 status at week 24 (n/%)
Total	20	0/0.00	7/35.00	20/100.00
Group A	7	0/0.00	6/85.71***	7/100.00
Group B	13	0/0.00	1/7.69***	13/100.00

1. IGA0/1 (clear/almost clear).

2. No patients in each group reached IGA0/1 at week 4, and all patients completed follow-up at week 24 reached IGA0/1 (only patients who completed 24 weeks of abrocitinib treatment were included in the analysis, per the study’s exclusion criteria).

3. ^***^P = 0.001.

#### 3.2.3 PP-NRS

PP-NRS scores exhibited a significant decline over time (overall P < 0.001). Baseline scores were comparable between the two groups (P = 0.485). Intergroup comparisons revealed that PP-NRS scores in Group A were significantly lower than those in Group B at week 4 (P = 0.019) and week 24 (P = 0.024), demonstrating the superiority of Group A in achieving rapid and sustained pruritus relief (**Table 2**).

#### 3.2.4 DLQI

DLQI scores showed significant improvement over time (overall P < 0.001). At baseline, the DLQI score in Group A was significantly higher than that in Group B (22.0 vs. 18.0, P < 0.001), with a 4-point difference, indicating more severe impairment in quality of life initially for Group A. However, no significant differences were observed in DLQI scores between the two groups at weeks 4, 12, and 24 (all P > 0.05), indicating that both groups achieved comparable quality of life outcomes following treatment (**Table 2**).

#### 3.2.5 Representative cases

**Figure 6:**
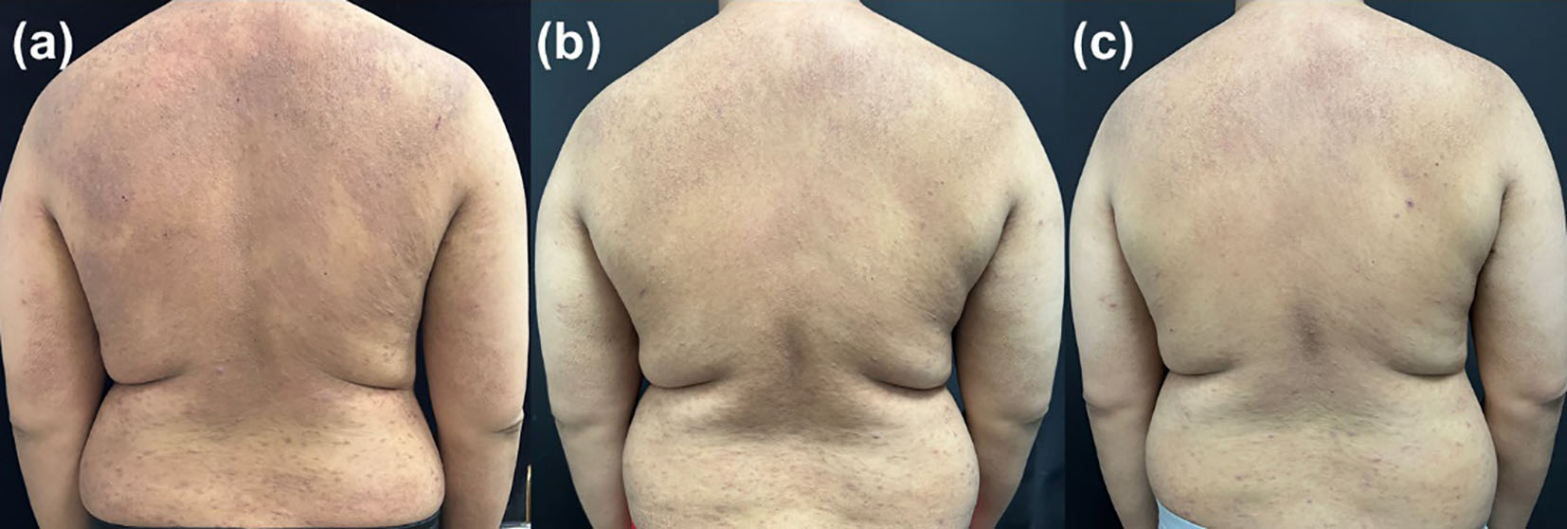
Representative clinical photographs of a patient in group B at **(A)** baseline, **(B)** week 4, **(C)** week 24.

To visually illustrate the clinical response patterns observed in the two treatment groups, representative pre- and post-treatment photographs of trunk lesions are presented in **Figures 5**, **6**.

**Figure 5** shows a patient from Group A (200 mg initiation strategy). At baseline, this patient presented with extensive erythematous plaques and severe excoriation (EASI 39.0, IGA 4, PP-NRS 10, DLQI 24). After 4 weeks of treatment, marked improvement in erythema and lesion clearance was already visible. By week 24, the skin lesions had nearly completely resolved, achieving an IGA score of 0.

**Figure 6** shows a patient from Group B (100 mg initiation strategy). At baseline, this patient presented with moderate-to-severe lesions (EASI 31.2, IGA 4, PP-NRS 8, DLQI 19). Although improvement was observed by week 4, the degree of response was less pronounced compared to the Group A patient at the same time point. By week 24, significant improvement was achieved, consistent with the quantitative findings.

These representative cases visually support the quantitative results: the 200 mg initiation strategy enabled more rapid disease control, particularly in patients with higher baseline disease burden, while the 100 mg initiation strategy ultimately achieved favorable outcomes by week 24.

### 3.3 Adverse events

During the treatment period, a total of 19 adverse events were reported in 13 patients (65%) (**Table 4**). All adverse events were mild to moderate in severity; no serious adverse events were reported.

**Table 4:** Adverse events.

Total number of AEs	19	Group A (11)	Group B (8)
Number of patients with AE	12 (60)	4	8
Gastrointestinal symptoms	5 (25)	2	3
Acne	4( 20)	2	2
Respiratory tract infections	3 (15)	2	1
Increased liver enzymes	2( 10)	2	0
Leukopenia	2 (10)	1	1
Herpes simplex infection	1 (5)	1	0
Headache	1 (5)	1	0
Fatigue	1 (5)	0	1

The incidence of adverse events in Group A was 71.43% (5/7), which was slightly higher than the 61.54% (8/13) observed in Group B. The most frequently reported adverse events included nausea (5 cases, 25%), acneiform eruption (4 cases, 20%), and respiratory tract infections (3 cases, 15%). Other reported events included elevated liver enzymes (2 cases, 10%), leukopenia (2 cases, 10%), headache (1 case, 5%), herpes simplex virus infection (1 case, 5%), and fatigue (1 case, 5%).

The vast majority of adverse events (15 cases, 83%) resolved spontaneously without intervention. Four patients improved following symptomatic management and continued abrocitinib therapy; these cases included two instances of acneiform eruption (treated with topical agents alone or in combination with oral minocycline), one instance of elevated liver enzymes (treated with oral hepatoprotective agents), and one instance of herpes simplex infection (managed with antiviral therapy). One patient with leukopenia recovered after medical treatment but ultimately discontinued abrocitinib and switched to dupilumab due to recurrent fluctuations in laboratory indices.

## 4 Discussion

Abrocitinib, a highly selective JAK1 inhibitor, has shown strong efficacy in multiple randomized controlled trials (RCTs). In the JADE MONO-1 and JADE MONO-2 trials, abrocitinib met its primary endpoints at week 12, with significant improvements in IGA 0/1 response and EASI-75 (11, 14). Another RCT comparing abrocitinib with dupilumab also demonstrated robust efficacy in pruritus relief and skin lesion improvement (15).

Several real-world studies have compared JAK inhibitors in AD. A meta-analysis of 63 real-world studies reported 16-week EASI-75 rates of 75% for abrocitinib, 51% for baricitinib, and 83% for upadacitinib (16). A comparative real-world study from Spain showed that JAK inhibitors produced faster early responses than biologics (17). A Korean cohort (n=66) reported EASI-75 and EASI-90 rates of 72.2% and 25.9% at week 16, with acne in 43.9% (18). A Japanese study found that JAK1 inhibitors had greater antipruritic effects than anti-IL-13 antibodies at 3 months (19). A Dutch upadacitinib study (n=48) reported IGA 0/1 in 47.9% at week 24, with acne-like eruptions in 25% (20). Another Dutch abrocitinib study (n=41) reported EASI-75 rates of 31.7% at week 12 and 41.5% at week 24 (21). These findings align with our results.

However, real-world data regarding the comparative effectiveness and safety of different abrocitinib dosage regimens remain relatively limited. A prospective real-world study from China (n=117) reported rapid disease improvement, with EASI-75 and EASI-90 rates of 74.3% and 50.5% at week 12 (22). However, that study did not compare different dosing strategies. In our study, we compared 200 mg step-down versus 100 mg step-up strategies in patients with moderate-to-severe AD. Our results indicate that the 200 mg initiation strategy offers superior effectiveness in terms of skin clearance (EASI, IGA) and pruritus relief (PP-NRS) with a manageable safety profile, thereby providing real-world evidence to guide clinical dosing decisions.

Compared to previous studies, our study offers three unique contributions. First, we directly compared two dynamic dosing strategies (200 mg step-down vs. 100 mg step-up), which has not been systematically evaluated before. Second, despite our cohort having more severe baseline disease (median IGA 4, all patients had prior systemic therapy, and 73% had prior dupilumab use), the 200 mg initiation group achieved an IGA 0/1 response rate of 85.71% at week 12. This rate is higher than the 72.2% EASI-75 rate reported in a Korean cohort (18) and the 75% rate reported in the meta-analysis (16), despite our patients having more severe baseline disease (median IGA 4). Third, the 200 mg group maintained superiority in EASI and PP-NRS scores through week 24, supporting sustained benefit of a higher starting dose. Consistent with RCTs, abrocitinib showed significant effectiveness in our study (11, 14). Notably, the 200 mg initiation group achieved an IGA 0/1 response rate of 85.71% at week 12, significantly higher than the 7.69% in the 100 mg group (P = 0.001), underscoring its potential in treating refractory AD.

Abrocitinib remained effective in patients who had failed prior JAK inhibitors (tofacitinib, upadacitinib, baricitinib) (23, 24). This may be due to differential JAK isoform selectivity, suggesting that switching between JAK inhibitors could be a viable strategy (25). Of the 20 patients in our cohort, 10 (50%) had failed prior systemic therapies. Among these, 6 out of 7 patients (85.7%) in the 200 mg group had prior treatment failure. This finding aligns with previous case reports showing that patients unresponsive to other therapies may still benefit from abrocitinib.

The two groups were well balanced across most baseline variables. However, baseline DLQI scores were significantly higher in Group A (22.0 vs. 18.0, P < 0.001), reflecting a clinical tendency for patients with greater quality of life impairment to receive the higher starting dose. The number of prior systemic therapies showed a marginal between-group difference (P = 0.057), suggesting that patients with more extensive prior treatment failure might also benefit from a higher initial dose.

Although baseline DLQI was higher in Group A, post-treatment DLQI scores showed no between-group difference. This suggests that patients with more severe quality of life impairment (DLQI ≥ 20) may be candidates for the 200 mg initiation strategy, while those with milder burden may achieve favorable outcomes with the 100 mg strategy by week 24. However, this threshold requires validation in larger studies.

Regarding safety, the AE incidence in our study was 65%, comparable to RCT rates (53%–73%). The most common AEs were nausea (25%) and acneiform eruption (20%). Acne incidence was slightly higher than in phase 3 trials (9.8% for 100 mg, 15.2% for 200 mg), possibly due to active inquiry in real-world practice. Most AEs were mild to moderate and resolved without intervention. No serious AEs occurred.

The relatively small sample size (n = 20) limits statistical power. This was an exploratory, real-world study with strict inclusion criteria, which limited the number of eligible patients. We addressed this by collecting data at four time points and conducting rigorous analyses. Even with this limitation, we observed significant between-group differences, suggesting large effect sizes. Additionally, as a single-center retrospective study, selection bias cannot be excluded. Given these limitations, our findings are preliminary and hypothesis-generating. Recent real-world studies with larger cohorts have begun to emerge. Future research should focus on identifying predictors of response, optimizing dose tapering protocols, and evaluating long-term safety.

## 5 Conclusion

In conclusion, this study validates the effectiveness and safety of abrocitinib in a real-world refractory AD population. The 200 mg starting dose offers advantages for rapid disease control, particularly in patients with high baseline disease burden. Larger prospective studies are warranted to confirm these findings.

## Data Availability

The original contributions presented in the study are included in the article/supplementary material. Further inquiries can be directed to the corresponding author/s.
